# A multi-reader comparison of normal-appearing white matter normalization techniques for perfusion and diffusion MRI in brain tumors

**DOI:** 10.1007/s00234-022-03072-y

**Published:** 2022-10-27

**Authors:** Nicholas S. Cho, Akifumi Hagiwara, Francesco Sanvito, Benjamin M. Ellingson

**Affiliations:** 1 Department of Radiological Sciences, David Geffen School of Medicine, University of California, Los Angeles, Los Angeles, CA USA; 2 UCLA Brain Tumor Imaging Laboratory (BTIL), Center for Computer Vision and Imaging Biomarkers, University of California, Los Angeles, Los Angeles, CA USA; 3 Department of Bioengineering, Henry Samueli School of Engineering and Applied Science, University of California, Los Angeles, Los Angeles, CA USA; 4 Medical Scientist Training Program, David Geffen School of Medicine, University of California, Los Angeles, Los Angeles, CA USA; 5 Department of Radiology, Juntendo University School of Medicine, Tokyo, Japan; 6 Unit of Radiology, Department of Clinical, Surgical, Diagnostic, and Pediatric Sciences, University of Pavia, Pavia, Italy; 7 Department of Neurosurgery, David Geffen School of Medicine, University of California, Los Angeles, Los Angeles, CA USA; 8 Department of Psychiatry and Biobehavioral Sciences, David Geffen School of Medicine, University of California, Los Angeles, Los Angeles, CA USA

**Keywords:** Normalized apparent diffusion coefficient, Normalized relative cerebral blood volume, Normal-appearing white matter, Diffusion MRI, Perfusion MRI, Glioma

## Abstract

**Purpose:**

There remains no consensus normal-appearing white matter (NAWM) normalization method to compute normalized relative cerebral blood volume (nrCBV) and apparent diffusion coefficient (nADC) in brain tumors. This reader study explored nrCBV and nADC differences using different NAWM normalization methods.

**Methods:**

Thirty-five newly diagnosed glioma patients were studied. For each patient, two readers created four NAWM regions of interests: (1) a single plane in the centrum semiovale (CSOp), (2) 3 spheres in the centrum semiovale (CSOs), (3) a single plane in the slice of the tumor center (TUMp), and (4) 3 spheres in the slice of the tumor center (TUMs). Readers repeated NAWM segmentations 1 month later. Differences in nrCBV and nADC of the FLAIR hyperintense tumor, inter-/intra-reader variability, and time to segment NAWM were assessed. As a validation step, the diagnostic performance of each method for IDH-status prediction was evaluated.

**Results:**

Both readers obtained significantly different nrCBV (*P* < .001), nADC (*P* < .001), and time to segment NAWM (*P* < .001) between the four normalization methods. nrCBV and nADC were significantly different between CSO and TUM methods, but not between planar and spherical methods in the same NAWM region. Broadly, CSO methods were quicker than TUM methods, and spherical methods were quicker than planar methods. For all normalization techniques, inter-reader reproducibility and intra-reader repeatability were excellent (intraclass correlation coefficient > 0.9), and the IDH-status predictive performance remained similar.

**Conclusion:**

The selected NAWM region significantly impacts nrCBV and nADC values. CSO methods, particularly CSOs, may be preferred because of time reduction, similar reader variability, and similar diagnostic performance compared to TUM methods.

**Supplementary Information:**

The online version contains supplementary material available at 10.1007/s00234-022-03072-y.

## Introduction

Relative cerebral blood volume (rCBV) values of brain tumors obtained from dynamic susceptibility contrast (DSC) perfusion MRI are routinely normalized (nrCBV) in both research and clinical settings to reduce variability across different MR protocols, scanners, and timepoints within the same patient. However, even though nrCBV values are affected by the chosen normalization technique itself [1, 2], there remains no consensus normalization method. Common normalization methods include placing a reference region of interest (ROI) on the contralateral normal-appearing white matter (NAWM), but numerous regions have been reported such as the white matter directly opposite to the tumor [3–5], the posterior limb of the internal capsule [6], the temporal lobe [7], and the centrum semiovale [8–12] along with variations in the placement of a single ROI [9] or multiple ROIs anteriorly to posteriorly [10, 11, 13]. Automated normalization methods, such as Gaussian-normalized nrCBV [1] and “standardized” nrCBV involving training-set data [1, 14–16], have also been described, but these methods require advanced software that limits their clinical feasibility.

There has also been growing interest in normalizing apparent diffusion coefficient (ADC) values of brain tumors obtained from diffusion MRI [5, 17–24]. For example, although ADC is a quantity measured in units (e.g., mm^2^/s), ADC values in a multicenter phase 2 trial of bevacizumab and chemotherapy in recurrent glioblastoma varied 7.3% in NAWM and 10.5% in cerebrospinal fluid across all sites [25]. Interestingly, ADC values of contralateral NAWM have also been shown to be significantly different across lobes in glioma patients [26], yet various NAWM normalization methods for normalized ADC (nADC) have been reported, including ROIs directly opposite to the tumor [5, 17], the posterior limb of the internal capsule [22], and the centrum semiovale [18, 19]. To our knowledge though, there remains no study comparing nADC normalization techniques in glioma patients.

The purpose of this reader study was to compare single-planar and multiple-spherical ROI NAWM normalization methods in the centrum semiovale and slice of the tumor center for nrCBV and nADC. In addition to assessing the impact of normalization methods on nrCBV and nADC values and reader variability, these normalization methods were validated by assessing their diagnostic performance when discriminating between IDH-wild-type gliomas and IDH-mutant 1p/19q-intact gliomas, since previous literature extensively showed the predictive value of ADC and nrCBV for this molecular profiling [21, 27, 28]. We hypothesized that there would be significantly different values for nrCBV and nADC based on the normalization method, and that the centrum semiovale and multiple-spherical ROI methods would provide significant benefit in reduced time compared to the tumor slice and single-planar ROI methods, respectively.

## Methods

### Patient selection

This study was conducted in compliance with the Health Insurance Portability and Accountability Act. All patients provided written informed consent to be part of our institutional review board approved clinical database (IRB no. 11-001427). In order to choose an adequate sample size for the present study, a power analysis based on previous findings [21] of nrCBV and nADC differences between IDH-wild-type and IDH-mutant 1p/19q intact gliomas was conducted using *β* = 0.8 and *α* = 0.05. Based on their nrCBV findings, a Cohen’s *d* effect size of 1.2 and a minimum number of 12 patients per group were determined; for their nADC findings, a Cohen’s *d* effect size of 1.4 and minimum number of 10 patients per group were determined.

Based on the results of the power analysis, a total of 18 IDH-wild-type glioma patients and 17 IDH-mutant 1p/19q intact glioma patients with histologically confirmed diagnoses and who obtained DSC-perfusion MRI, diffusion MRI, and anatomical MRI scans before treatment were retrospectively studied. Since the IDH-mutational status assessment was not the focus of the study but rather performed as a benchmark for the validation of the normalization methods, IDH-mutant 1p/19q co-deleted tumors (oligodendrogliomas) were not included since previous literature already showed that the usefulness of ADC and rCBV to detect this tumor type is limited because of their intermediate features between IDH-mutant 1p/19q intact gliomas and IDH-wild type gliomas [27]. IDH mutation was assessed by immunohistochemistry, genomic sequencing analysis, and/or polymerase chain reaction [29], and 1p/19q codeletion status was determined using fluorescence in situ hybridization. Patient scans were conducted between August 2015 and October 2019. Patient data are summarized in Table 1.

**Table 1:** Clinical data of patients

Characteristic	Patients (*n* **= **35)
Average age (years) ± SD	48 ± 16
Sex (male/female)	24/11
Tumor location	
Hemisphere (left/right)	16/19
Frontal lobe	9
Frontotemporal lobes	2
Temporal lobe	11
Temporoparietal lobes	2
Parietal lobe	7
Parieto-occipital lobes	1
Occipital lobe	2
Thalamus	1
Tumor grade (2/3/4)	9/13/13
IDH mutation status (wild type/mutant)	18/17

### Image acquisition and processing

Anatomical, diffusion, and DSC perfusion MRI were obtained on 1.5T or 3T MRI scanners (Siemens Healthcare; Erlangen, Germany). Anatomical MRI and diffusion-weighted imaging (DWI) were collected according to the international standardized brain tumor imaging protocol (BTIP) [30]. ADC maps were calculated from either DWI or diffusion tensor imaging (DTI) data with *b*-values of 0 and 1000 s/mm^2^. For DSC perfusion MRI, images were collected according to previously described single-echo and multi-echo imaging protocols [31–33]. DSC data were first motion corrected using FSL (*mcflirt*; Functional Magnetic Resonance Imaging of the Brain Software Library; Oxford, England), and a bidirectional contrast agent leakage correction method was used to calculate rCBV maps [34]. All parameter maps were registered to the post-contrast T1-weighted images (1-mm isotropic resolution) using a six-degree-of-freedom rigid transformation and a mutual information cost function using FSL software (*flirt*).

### Normal appearing white matter and tumor segmentation

**Fig. 1:**
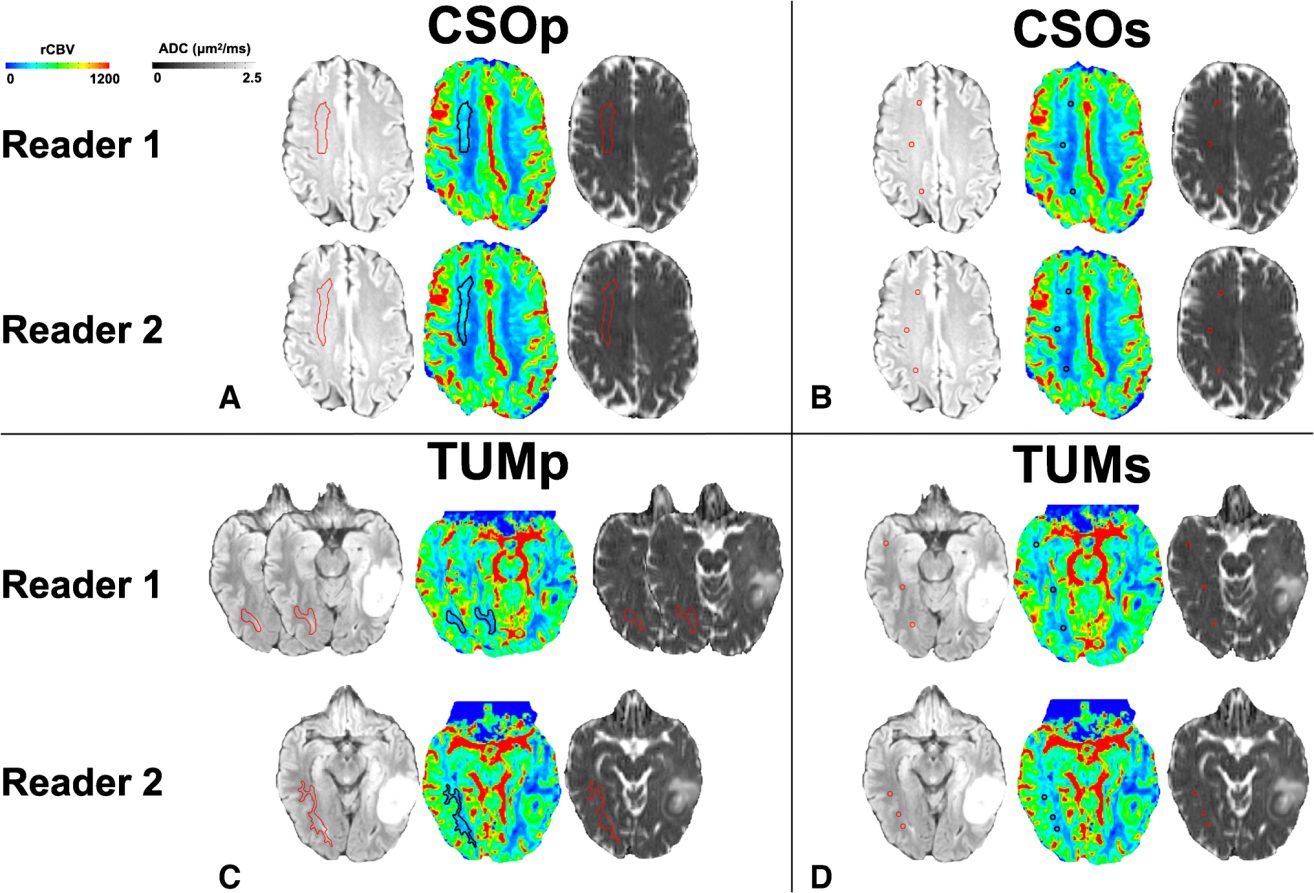
Example NAWM segmentations from both readers. NAWM segmentations using **A** the planar method in the centrum semiovale (CSOp), **B** spherical method in the centrum semiovale (CSOs), **C** planar method in the slice contralateral to the center of tumor (TUMp), and **D** spherical method in the slice contralateral to the center of tumor (TUMs) on T2/FLAIR images and rCBV and ADC maps. NAWM, normal-appearing white matter; rCBV, relative cerebral blood volume; ADC, apparent diffusion coefficient

**Fig. 2:**
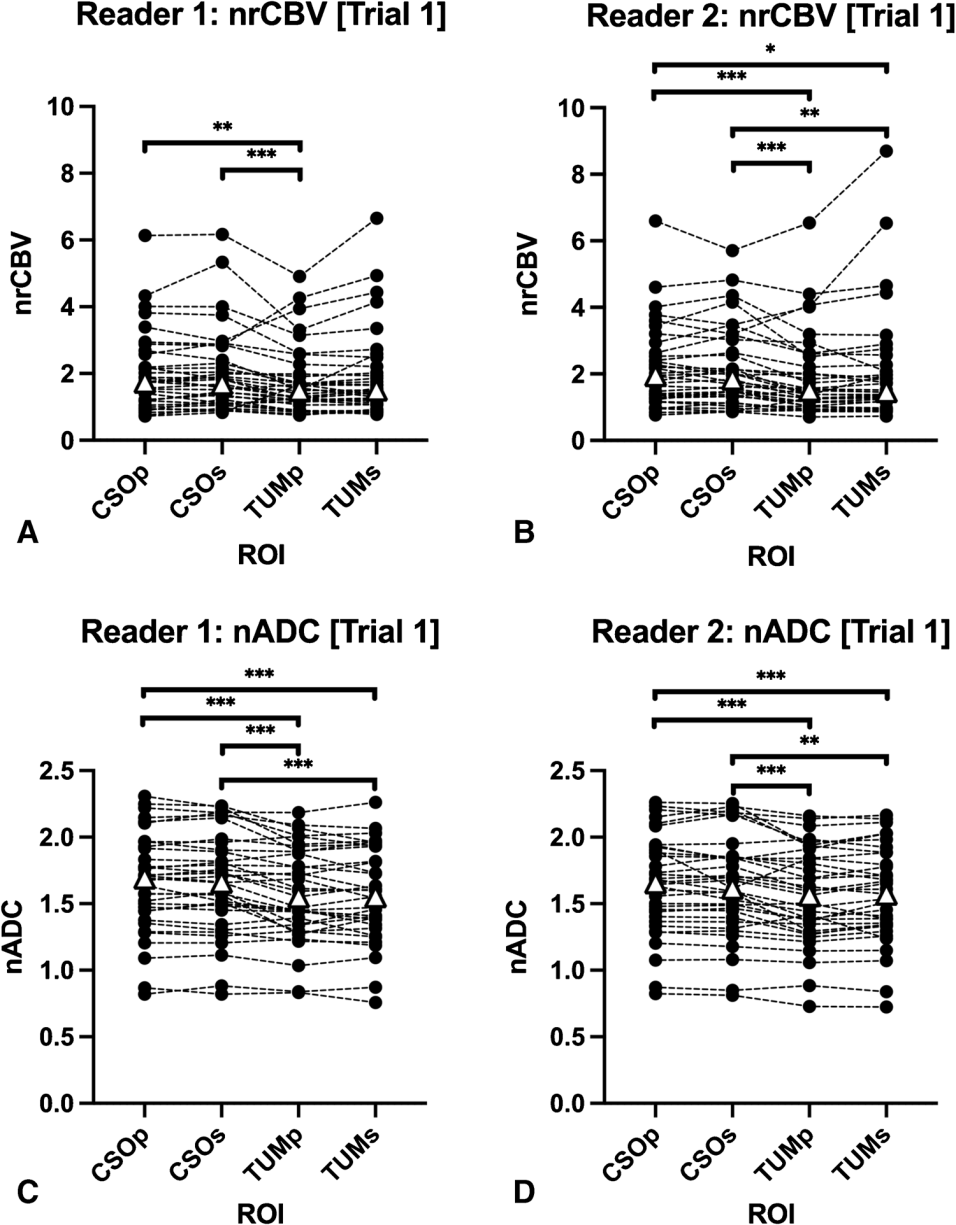
Differences in nrCBV and nADC based on normalization method (trial 1). Post-hoc analyses revealed significant differences for **A/B** nrCBV and **C/D** nADC between centrum semiovale (CSO) and tumor slice (TUM) methods for both readers in trial 1, but not between planar (p) and spherical (s) methods within the same normalization region. △ indicates median; * indicates *P* < .05; ** indicates *P* < .01; *** indicates *P* < .001. nrCBV, normalized relative cerebral blood volume; nADC, normalized apparent diffusion coefficient

**Fig. 3:**
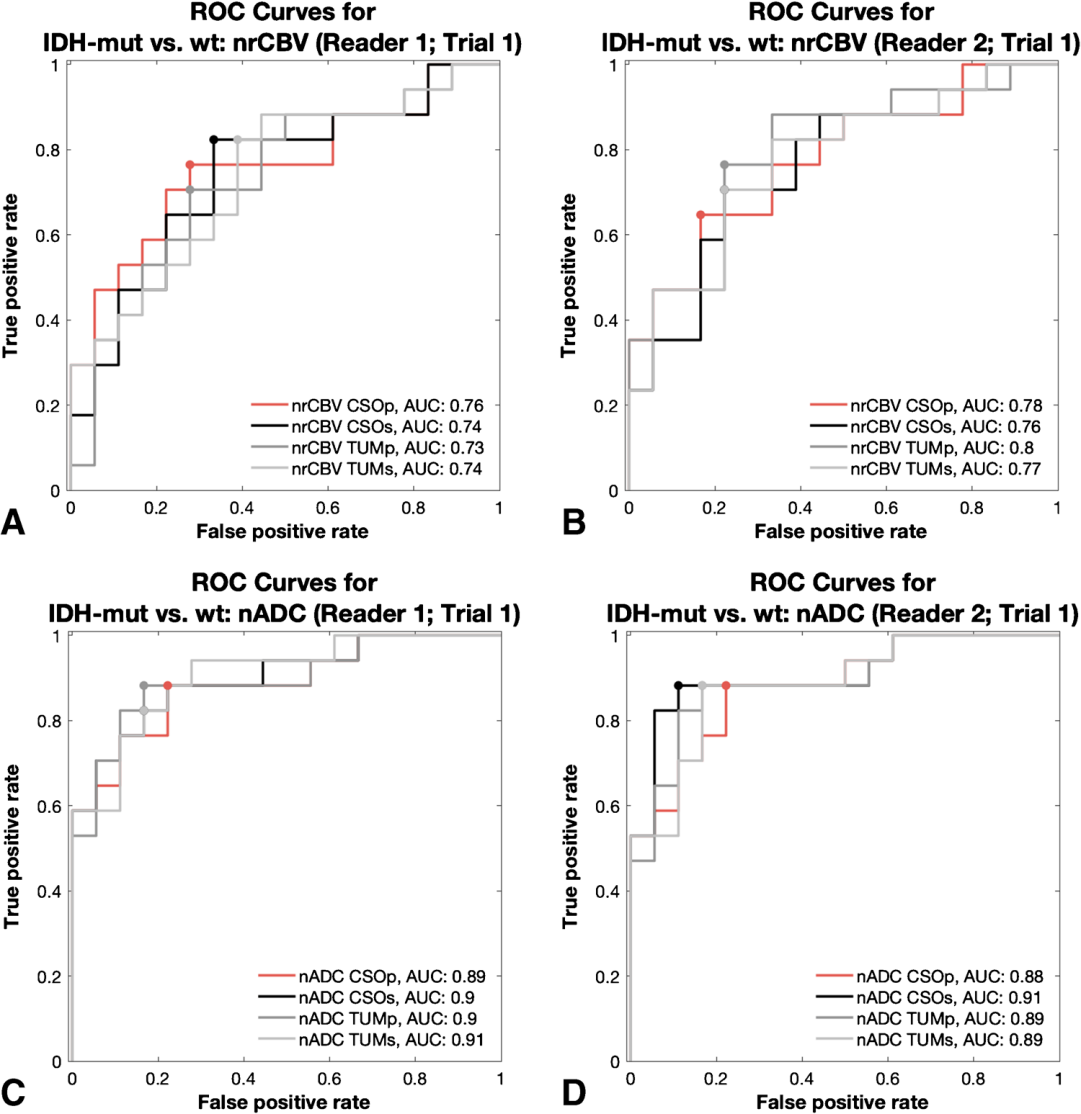
Receiver-operating characteristic (ROC) curves for predicting IDH mutation status (trial 1). Different normalization methods resulted in similar area under the curve values for IDH-mutation status prediction using **A**/**B** nrCBV and **C**/**D** nADC. nrCBV, normalized relative cerebral blood volume; nADC, normalized apparent diffusion coefficient

The two readers in this study were a board-certified radiologist (AH) and a radiology resident (FS) with 10 and 6 years of experience in neuroimaging analysis, respectively. Both readers were blinded to patient information, and each reader segmented four contralateral NAWM ROIs using ITK-SNAP software (http://www.itksnap.org/) [35] that avoided cortex, large vessels, and ventricles with the following names and instructions (see supplementary information for full reader instructions): (1) CSOp, a planar ROI of 400–450 mm^2^ drawn on a single slice in the contralateral centrum semiovale approximately 3 mm (~3 slices) superior to the lateral ventricles similar to Conte et al. [9] (Fig. 1A); (2) CSOs: 3 intra-slice 3D spheres of 5 mm diameter (~5 slices) spanning anteriorly to posteriorly in the contralateral centrum semiovale approximately 3 mm superior to the lateral ventricles as done in prior studies [11, 18] and similar to Smits et al. [10] (Fig. 1B); (3) TUMp: a planar ROI of 400–450 mm^2^ drawn on a single slice in the slice of the center of the tumor as similarly suggested by the Quantitative Imaging Biomarkers Alliance’s (QIBA’s) Stage 2 Consensus Profile guidelines for nrCBV [3] (Fig. 1C); and (4) TUMs: 3 intra-slice 3D spheres of 5 mm diameter (~5 slices) spanning anteriorly to posteriorly in the slice of the center of the tumor (Fig. 1D). If a contiguous single-slice planar ROI was unable to be created, readers were allowed to create 2 planar ROIs on 2 consecutive slices (Fig. 1C). One month later, the patient order was randomized, and each reader repeated NAWM ROI segmentations and recorded the time needed to segment each ROI. nrCBV and nADC maps were calculated by dividing the rCBV and ADC maps by the mean rCBV and ADC values of the NAWM ROIs. A volume of interest (VOI) was segmented on the FLAIR hyperintense tumor using an in-house, semi-automated thresholding method using the Analysis of Functional NeuroImages (AFNI) software (NIMH Scientific and Statistical Computing Core; Bethesda, MD, USA; https://afni.nimh.nih.gov) [36]. Median nrCBV and nADC values of the FLAIR hyperintense tumor were derived using each of the 4 normalization techniques.

### Statistical analysis

All calculations and analyses were performed in MATLAB (Release 2020a, MathWorks, Natick, MA, USA) or GraphPad Prism software (Version 8.4 GraphPad Software, San Diego, California). The D’Agostino and Pearson test was conducted to assess if data were normally distributed and to apply appropriate parametric or nonparametric statistical methods. To assess intra-reader differences in nrCBV, nADC, and the time to create NAWM ROIs based on the normalization method, the repeated-measures ANOVA test with post hoc Tukey’s multiple comparisons tests and the Friedman test with post hoc Dunn’s multiple comparisons tests were performed for normally and non-normally distributed data, respectively. The intraclass correlation coefficient (ICC) (2, 1) model was used to assess inter-reader reproducibility of nrCBV and nADC from each normalization method at each trial, and the ICC (3, 1) model was used to assess intra-reader repeatability of nrCBV and nADC of each normalization method between trials [37]. Because ICC analyses require normally distributed data [38], the Box-Cox transformation was first performed on non-normally distributed data, and the transformed, normally distributed data was used for ICC analyses. In order to validate the nrCBV and nADC values obtained from each normalization method, receiver-operating characteristic (ROC) curve analyses were performed to assess the IDH-mutational status predictive performance of the nrCBV and nADC values obtained from different normalization methods. Significance level was set to *α* = 0.05.

## Results

Full reporting of this study’s results, including figures for trial 2 results, is presented in the supplementary information. Normality tests demonstrated that nADC data were normally distributed, while nrCBV and time to create NAWM ROI’s data were non-normally distributed, so appropriate parametric and nonparametric statistical methods were chosen for each metric.

For each trial, each reader obtained overall significantly different nrCBV (*P* <. 001) and nADC (*P* < .001) values between the four normalization methods (Figs. 2 and S1). In post-hoc analyses, there were significant differences in nrCBV and nADC between CSO and TUM normalization methods (Table S1; Figs. 2 and S1). For example, when comparing CSO and TUM methods in trial 1, the median difference in nrCBV and mean difference in nADC ranged in magnitude between 0.10–0.27 and 0.07–0.09, respectively (Table S1). However, there were no significant differences in nrCBV or nADC between planar and spherical methods within the same normalization region (CSOp vs. CSOs or TUMp vs. TUMs). For these comparisons, the median difference in nrCBV and mean difference in nADC in trial 1 was greatly reduced to magnitudes ranging between 0.02–0.05 and 0.002–0.001, respectively (Table S1). ICC analyses indicated that each normalization method had excellent reproducibility (*r* > 0.90 as stated by Koo et al. [37]) between readers (Table 2; Fig. S2) and within readers when they repeated NAWM segmentations after 1 month (Table 2; Fig. S3). As a validation step, ROC curve analyses for IDH-mutation status prediction revealed that the nrCBV and nADC values yielded similar area under the curve values regardless of normalization method (Figs. 3 & S4; trial 1 AUC for *nrCBV* = 0.73–0.80; AUC for *nADC* = 0.88–0.91).

**Table 2:** Inter-reader reproducibility between readers and intra-reader repeatability between trials for nrCBV and nADC

MRI metric (NAWM)	Inter-reader reproducibility ICC (95% CI) for trials 1/2	Intra-reader repeatability ICC (95% CI) for readers 1/2
nrCBV (CSOp)	0.97 (0.79–0.99)/0.95 (0.90–0.97)	0.99 (0.98–1)/0.99 (0.98–0.99)
nrCBV (CSOs)	0.96 (0.86–0.99)/0.94 (0.80–0.98)	0.98 (0.96–0.99)/0.98 (0.96–0.99)
nrCBV (TUMp)	0.94 (0.87–0.97)/0.97 (0.95–0.99)	0.98 (0.96–0.99)/0.99 (0.98–0.99)
nrCBV (TUMs)	0.95 (0.90–0.97)/0.92 (0.85–0.96)	0.98 (0.95–0.99)/0.99 (0.98–0.99)
nADC (CSOp)	0.99 (0.99–1)/1 (0.99–1)	1 (0.99–1)/1 (1–1)
nADC (CSOs)	0.99 (0.98–0.99)/0.98 (0.95–0.99)	0.99 (0.99–1)/0.99 (0.98–1)
nADC (TUMp)	0.97 (0.94–0.99)/0.97 (0.93–0.98)	0.99 (0.98–0.99)/0.99 (0.97–0.99)
nADC (TUMs)	0.97 (0.95–0.99)/0.98 (0.96–0.99)	0.99 (0.98–0.99)/0.99 (0.97–0.99)

**Fig. 4:**
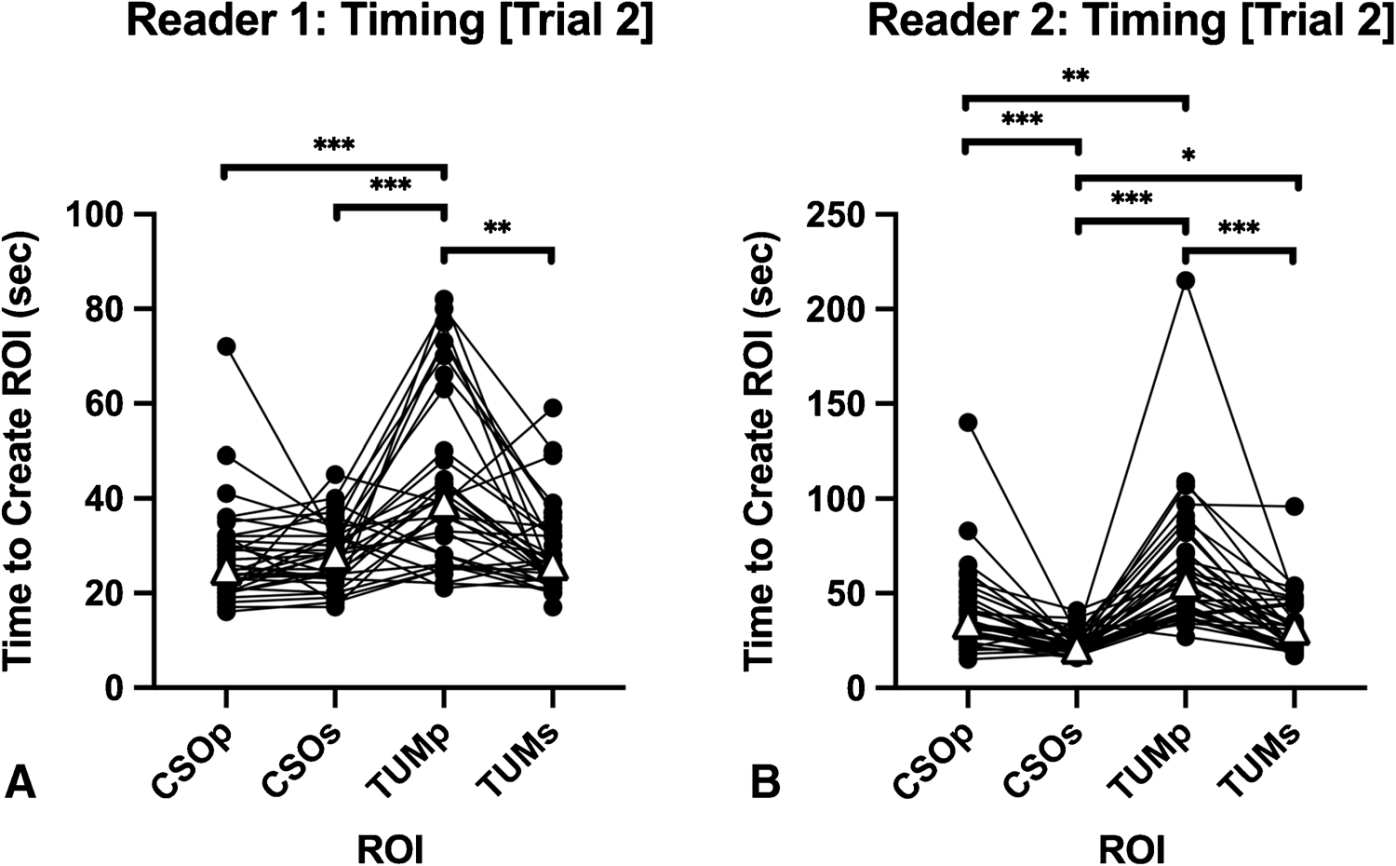
Time to create NAWM ROIs. Significant differences in the time to create each ROI were observed. Broadly, CSO methods were quicker than TUM methods, and spherical methods were quicker than planar methods. △ indicates median; * indicates *P* < .05; ** indicates *P* < .01; *** indicates *P* < .001. NAWM, normal-appearing white matter; ROI, region of interest; CSO, centrum semiovale; TUM, tumor

There were significant differences in the times to create each ROI (*P* < .001; Table S2; Fig. 4). In general, CSO methods were quicker than TUM methods, particularly for the planar method (median time savings of CSOp vs. TUMp = 11 s, *P* < .001 for reader 1; 16 s, *P* < .01 for reader 2), and the spherical method was quicker than the planar method, particularly for the TUM region (median time savings of TUMs vs. TUMp = 11 s, *P* < .01 for reader 1; 23 s, *P* < .001 for reader 2).

## Discussion

The primary finding of the present study was that nrCBV and nADC values were significantly different based on the NAWM region, but not based on planar or spherical methods within the same NAWM region. As a result, the present findings support that it is imperative to be consistent in ROI-based normalization methods for both nrCBV and nADC. This study adds to previous literature by showing that nrCBV is significantly different based on NAWM normalization methods [1, 2] and, to our knowledge, being the first to demonstrate differences in nADC in glioma patients based on NAWM normalization method, which are in line with previous findings that ADC is significantly different across contralateral NAWM regions in glioma patients [26]. In order to increase reproducibility and to better guide threshold-based interpretations of nrCBV and nADC across institutions, it is critical for research studies to describe the anatomical location and size of the NAWM ROI for rCBV and ADC normalization as done in some studies [9–11, 13, 18, 19]. For example, the excellent inter-reader reproducibility for nADC observed in the present study is in line with the high ICC values for nADC by two readers in a study by Thust et al., where the authors explicitly stated that each rater segmented NAWM ROIs in the CSO with similar volume to the tumor [19]. Furthermore, providing additional detail on the selection of a specific slice of the target NAWM region (e.g., CSO ~3 mm above the lateral ventricles in the present study) as done by Smits et al. and Cho et al. for nrCBV [10, 11] and Hagiwara et al. for nADC [18] may reduce variability and subjectivity in the normalization process.

All four normalization methods in the present study had similar intra-reader repeatability and inter-reader reproducibility as well as IDH-mutation status predictive performance, but there were significant reductions in time when performing the CSO methods compared to the TUM methods. One likely explanation for the increased time to create NAWM ROIs in TUM regions was for cases where the tumor was located in regions with minimal contralateral white matter, such as near subcortical structures or in the temporal lobes (Fig. 1C and D), so delineating a NAWM ROI that avoids gray matter, normal vessels, and ventricles was particularly challenging. Moreover, selecting a NAWM ROI in the tumor slice of those regions would be even more challenging if there was bilateral tumor infiltration. As a result, the present findings may support the usage of the centrum semiovale [8–11, 18] as a target NAWM region instead of the white matter directly opposite the tumor [3, 4, 17] because the centrum semiovale is reliably a large region of white matter that is easily identifiable to neuroradiologists and research lab members alike, as also similarly stated by Thust et al. [19]. Of note, the current guidelines provided by QIBA’s Stage 2 Consensus Profile for nrCBV propose a > 2 × 2 cm TUMp NAWM ROI [3]. The present results of a similar 400–450 mm^2^ TUMp ROI—which the study authors proposed given the difficulty of creating a 2 cm ROI in certain tumor regions described above—suggest that although tumor-slice ROIs provide similar diagnostic performance, intra-reader repeatability, and inter-reader reproducibility compared to CSO ROIs, CSO NAWM methods may be better options in terms of time efficiency and ease.

Additionally, both readers had significantly reduced times creating TUMs ROIs compared to TUMp ROIs, and one reader had significantly reduced times creating CSOs ROIs compared to CSOp ROIs. The time reduction for the spherical methods compared to the planar methods may also be explained by similar reasons of ease. A significant advantage of the separable, spherical method is that it could be easier to avoid gray matter, vessels, and ventricles compared to a contiguous, planar method, especially in research settings in which lab members who are not radiologists may be involved. Furthermore, if the tumor is bilateral, spherical methods may be easier to avoid the lesion compared to planar methods. However, 3D ROIs may not be able to be created in clinical software, so the CSOp method may be preferred in clinical settings when assessing quantitative maps generated from the scanner or from clinical software products. An alternative to the 3D CSOs method in the present study could also be placing 2D circular ROIs in the CSO as done by Smits et al. [10] to allow for use in clinical settings. One key difference between the study by Smits et al. and the present study was that the former placed their 2D planar ROIs on the original rCBV maps with a large 5 mm slice thickness, while in the present study, all ROIs were placed on rCBV and ADC maps registered to the post-contrast T1-weighted image with 1 mm slice thickness. Future studies may want to investigate differences in nrCBV and nADC normalization-based 2D and 3D ROIs along with consideration of slice thickness.

Although NAWM normalization techniques are popular for nrCBV, it is worth mentioning other normalization approaches applied in some other studies. For example, there has also been interest in the automatic normalization, or “standardization,” of rCBV parametric maps by transforming rCBV values to a standardized scale, precluding the need for manual NAWM ROIs [1, 14–16]. Standardization of rCBV has been shown to reduce variability compared to manual NAWM methods [1, 14, 15], though standardized rCBV metrics had similar performance with NAWM-based nrCBV metrics for assessing post-treatment tumor burden in stereotactic biopsy samples of recurrent high-grade glioma [16]. Additionally, standardization requires the use of a training data set for each anatomical region and MRI protocol, which may explain why NAWM methods remain popular in research studies involving rCBV analyses. Intra-scan, nonmanual rCBV normalization techniques—such as min-max normalization [39], normalized by the standard deviation across the whole brain [1] and normalized by the mean values outside the tumor [40]—have also been reported in limited cases. Some studies have also utilized normalization ROIs that include solely gray matter or a combination of gray and white matter [24, 41–43], likely on the basis that gliomas can infiltrate into gray matter. However, gray matter normalization has been demonstrated to cause systematic differences in tumor nrCBV compared to previously reported nrCBV thresholds obtained by the more conventional NAWM methods [24]. As a result, the present study did not explore automated or gray matter normalization methods given their limited use, and instead, the present study aimed to assess variability within readers based on previously reported NAWM techniques in the literature.

It is also important to note that the usage of nADC remains controversial. Absolute ADC values are measured in units (e.g., mm^2^/s), so normalization may not be justified. Additionally, there have been mixed findings on the potential benefit of nADC over ADC in glioma patients [17–21]. Nevertheless, ADC values of NAWM and cerebrospinal fluid have been reported to vary across patients in a multicenter trial [25], which may support the increased implementation of nADC in the future. As a result, the characterization of various NAWM methods for nADC in the present study remains valuable as the potential clinical utility of nADC continues to be investigated.

This study has several limitations. Although the present study’s sample size of IDH-mutant and IDH-wild-type gliomas was chosen based on power analysis using previously reported [21] nADC and nrCBV differences between IDH status, there still remains the possibility that the sample size was limited. Nevertheless, significant differences in nrCBV and nADC were observed between CSO and TUM normalization methods, and ROC analyses of nrCBV and nADC remained highly predictive of IDH status within our study cohort, so we believe that the study size is justified. Increasing the sample size, though, may potentially allow for better capturing the heterogeneity of brain tumors, such as tumor location and the presence of bilateral infiltration, which can lead to potential challenges during NAWM normalization. Additionally, one patient was scanned at 1.5T. However, CBV and ADC are theoretically independent of field strength [44]. Furthermore, we believe including this patient is acceptable given that we assessed intra-patient, paired differences based on NAWM techniques, and that ADC and rCBV NAWM normalization should compensate for field strength differences [22], if any. This study was also limited to datasets acquired from a single institution. A multicenter study may be valuable to better assess intra-reader repeatability and inter-reader reproducibility of the ROI-based normalization methods used in this study. Finally, automated normalization methods were not compared to the multiple ROI-based normalization methods in this study, but these approaches may provide for faster and more reproducible results.

## Conclusion

There can be significant differences in nrCBV and nADC values depending on the selected NAWM region. CSOs normalization may be useful in research settings, while CSOp normalization may be useful in clinical settings. Studies involving normalized MRI metrics based on ROI methods should clearly state the anatomical region, size, and approximate slice location of the normalization ROI to improve reproducibility and data interpretation for outside institutions.

## Supplementary information

High resolution image (PDF 4.92 MB)

## Abbreviations

CSOp: Centrum semiovale, 2D planar method
CSOs: Centrum semiovale, 3D spherical method
IDH: Isocitrate dehydrogenase
nADC: Normalized apparent diffusion coefficient
NAWM: Normal-appearing white matter
nrCBV: Normalized relative cerebral blood volume
QIBA: Quantitative Imaging Biomarkers Alliance
rCBV: Relative cerebral blood volume
ROI: Region of interest
VOI: Volume of interest
TUMp: Tumor slice, 2D planar method
TUMs: Tumor slice, 3D spherical method
