# Supplementary material for: A multi-reader comparison of normal-appearing white matter normalization techniques for perfusion and diffusion MRI in brain tumors
Source: Neuroradiology. 2022 Oct 27;65(3):559–68. doi: 10.1007/s00234-022-03072-y (PMC9905164; doi:10.1007/s00234-022-03072-y)
Supplement: Supplementary file 1 — High resolution image (PDF 4.92 MB) [file 234_2022_3072_MOESM1_ESM.pdf]

A Multi-Reader Comparison of Normal Appearing White Matter Normalization Techniques for Perfusion and Diffusion MRI in Brain Tumors

Nicholas S. Cho[1-4], Akifumi Hagiwara[1,2,5], Francesco Sanvito[1,2,6], Benjamin M. Ellingson[1-3,7,8*]

[1] Department of Radiological Sciences, David Geffen School of Medicine, University of California, Los Angeles, Los Angeles, CA, USA

[2] UCLA Brain Tumor Imaging Laboratory (BTIL), Center for Computer Vision and Imaging Biomarkers, University of California, Los Angeles, Los Angeles, CA, USA

[3] Department of Bioengineering, Henry Samueli School of Engineering and Applied Science, University of California Los Angeles, Los Angeles, CA, USA

[4] Medical Scientist Training Program, David Geffen School of Medicine, University of California, Los Angeles, Los Angeles, CA, USA.

[5] Department of Radiology, Juntendo University School of Medicine, Tokyo, Japan

[6] Unit of Radiology, Department of Clinical, Surgical, Diagnostic, and Pediatric Sciences, University of Pavia, Pavia, Italy

[7] Department of Neurosurgery, David Geffen School of Medicine, University of California, Los Angeles, Los Angeles, CA, USA

[8] Department of Psychiatry and Biobehavioral Sciences, David Geffen School of Medicine, University of California, Los Angeles, Los Angeles, CA, USA

Supplementary Tables

Table S1. Detailed post-hoc statistical results of nrCBV and nADC differences.

nrCBV (Non-parametric; Dunn's test)			nADC (Parametric; Tukey's test)		
**Post-hoc Test Based on NAWM Method**	**Reader 1: Rank Sum Difference (Median Difference) for Trials 1 / 2**	**Reader 2: Rank Sum Difference (Median Difference) for Trials 1 / 2**	**Post-hoc Test Based on NAWM Method**	**Reader 1: Mean Difference (95% CI) for Trials 1 / 2**	**Reader 2: Mean Difference (95% CI) for Trials 1 / 2**
**CSOp vs. CSOs**	-9 (-0.03) / 1 (0.04)	-6 (-0.03) / -16 (-0.13)	**CSOp vs. CSOs**	0.002 (-0.02–0.02) / 0.02 (-0.02–0.06)	0.01 (-0.02–0.04) / 0.02 (-0.01–0.05)
**CSOp vs. TUMp**	36 (0.17)** / 39 (0.23)**	41 (0.21)*** / 35 (0.22)**	**CSOp vs. TUMp**	0.08 (0.03–0.13)*** / 0.08 (0.04–0.13)***	0.09 (0.05–0.13)*** / 0.08 (0.04–0.13)***
**CSOp vs. TUMs**	19 (0.10) / 30 (0.11)*	31 (0.21)* / 19 (0.14)	**CSOp vs. TUMs**	0.09 (0.04–0.14)*** / 0.09 (0.04–0.13)***	0.08 (0.04–0.12)*** / 0.07 (0.03–0.10)***
**CSOs vs. TUMp**	45 (0.21)*** / 38 (0.22)**	47 (0.27)*** / 51 (0.29)***	**CSOs vs. TUMp**	0.08 (0.03–0.13)*** / 0.06 (0.02–0.11)**	0.08 (0.03–0.13)*** / 0.06 (0.02–0.10)**
**CSOs vs. TUMs**	28 (0.16) / 29 (0.20)*	37 (0.22)** / 35 (0.24)**	**CSOs vs. TUMs**	0.09 (0.04–0.13)*** / 0.07 (0.02–0.11)**	0.07 (0.02–0.11)** / 0.04 (0.01–0.08)*
**TUMp vs. TUMs**	-17 (-0.05) / -9 (-0.09)	-10 (-0.02) / -16 (-0.08)	**TUMp vs. TUMs**	0.006 (-0.03–0.04) / 0.002 (-0.02–0.03)	-0.01 (-0.04–0.02) / -0.02 (-0.06–0.02)

* indicates $P < .05$; ** indicates $P < .01$; *** indicates $P < .001$

Table S2. Detailed post-hoc statistical results of differences in times to create ROIs.

Time to Create ROIs (Non-Parametric; Dunn's test)		
**Post-hoc Test Based on NAWM Method**	**Reader 1: Rank Sum Difference (Median Difference in Seconds) for Trial 2**	**Reader 2: Rank Sum Difference (Median Difference in Seconds) for Trial 2**
**CSOp vs. CSOs**	-7 (-1)	43.5 (11)***
**CSOp vs. TUMp**	-51 (-11)***	-38.5 (-16)**
**CSOp vs. TUMs**	-16 (-2)	15 (5)
**CSOs vs. TUMp**	-44 (-11)***	-82 (-28)***
**CSOs vs. TUMs**	-9 (-2)	-28.5 (-4)*
**TUMp vs. TUMs**	35 (11)**	53.5 (23)***

* indicates $P < .05$; ** indicates $P < .01$; *** indicates $P < .001$

Supplementary Figures

Figure S1. Differences in nrCBV and nADC based on normalization method (Trial 2).

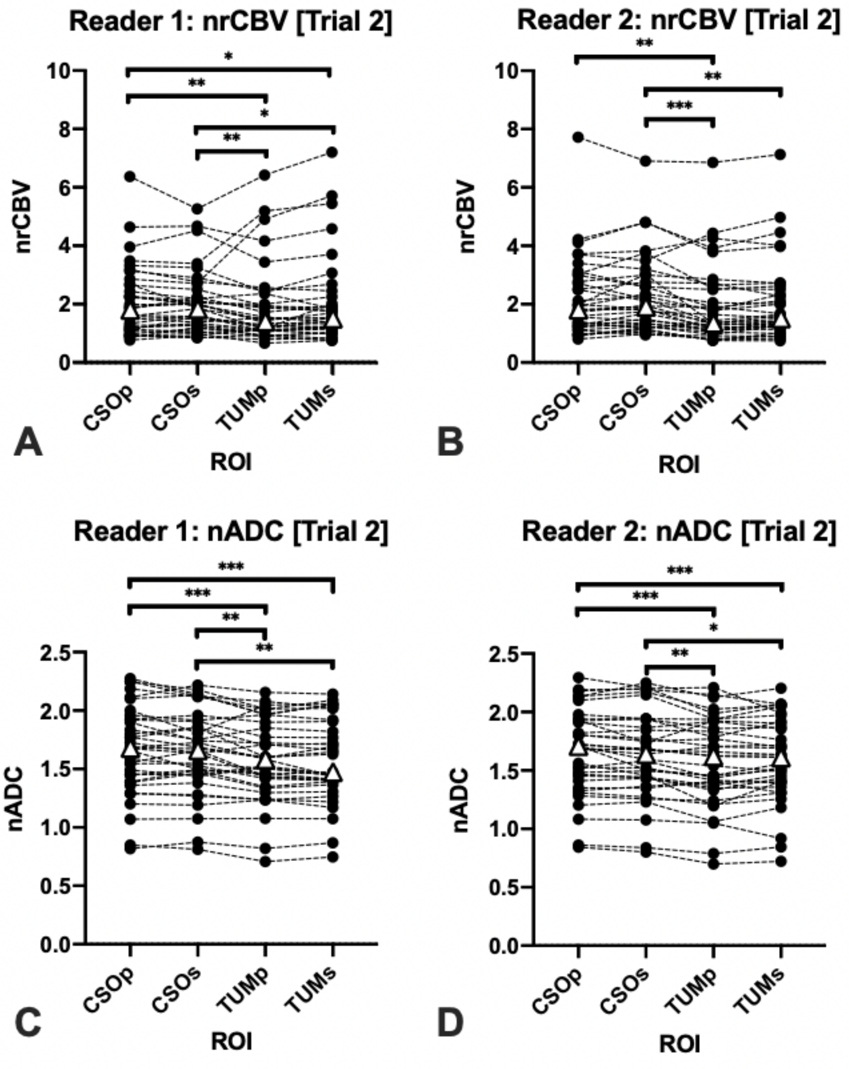

Figure S1. Differences in nrCBV and nADC based on normalization method (Trial 2). Post-hoc analyses revealed significant differences for **A/B**) nrCBV and **C/D**) nADC between centrum semiovale (CSO) and tumor slice (TUM) methods for both readers in Trial 2, but not between planar (p) and spherical (s) methods within the same normalization region, similar to results from Trial 1. △ indicates median; * indicates $P < .05$; ** indicates $P < .01$; *** indicates $P < .001$

Figure S2. ICC analyses between readers (Trials 1 & 2).

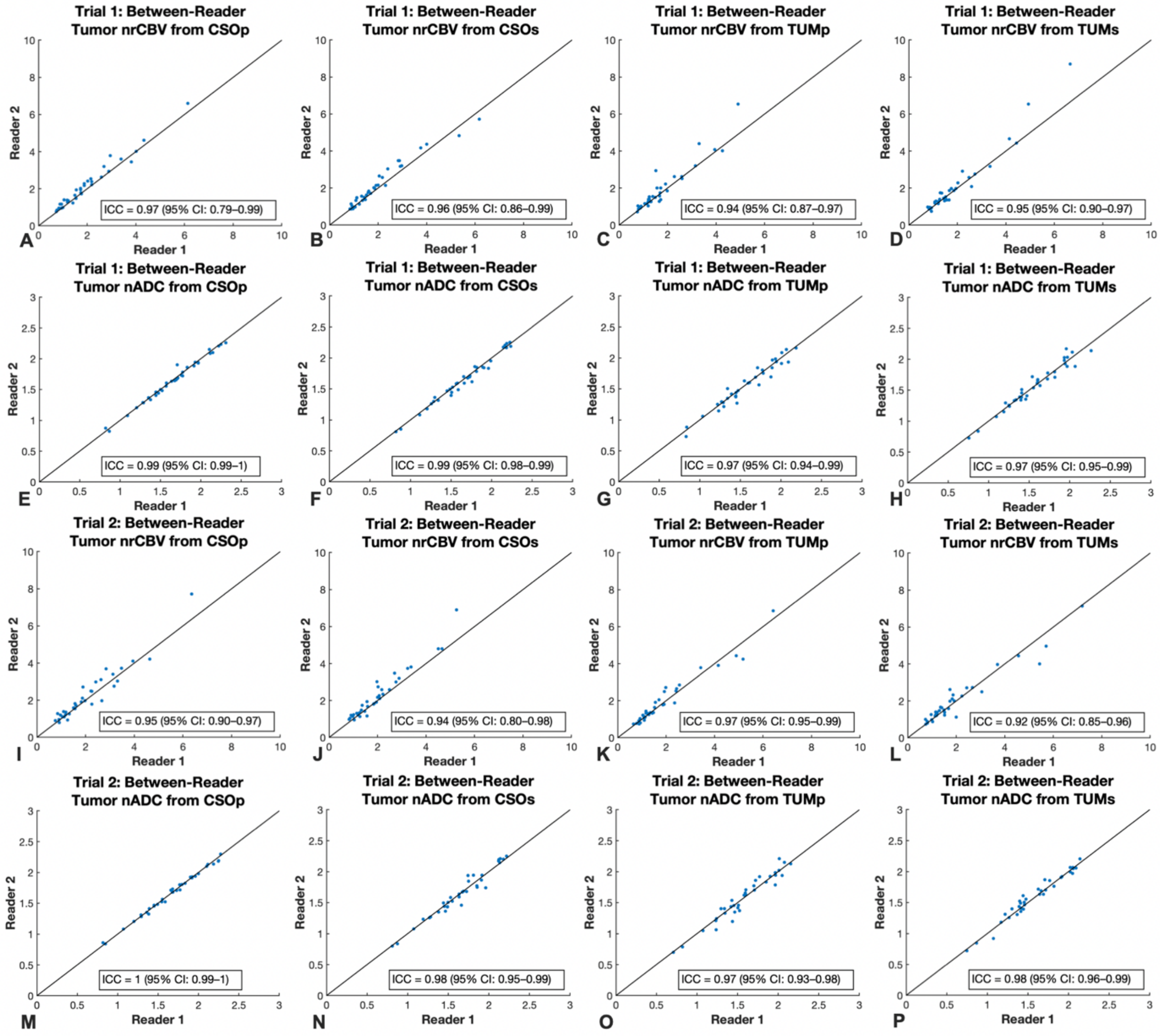

Figure S2. ICC analyses between readers (Trials 1 & 2). nrCBV and nADC from **A/E/I/M**) CSOp, **B/F/J/N**) CSOs, **C/G/K/O**) TUMp, and **D/H/L/P**) TUMs displayed excellent (r>0.9) reproducibility between readers in both Trials 1 and 2. Because nrCBV data were non-normally distributed, all plots of ICC analyses display the non-transformed data points with the appropriate ICC statistical results from the normally distributed data following Box-Cox transformation.

Figure S3. ICC analyses of intra-reader repeatability across Trials 1 & 2

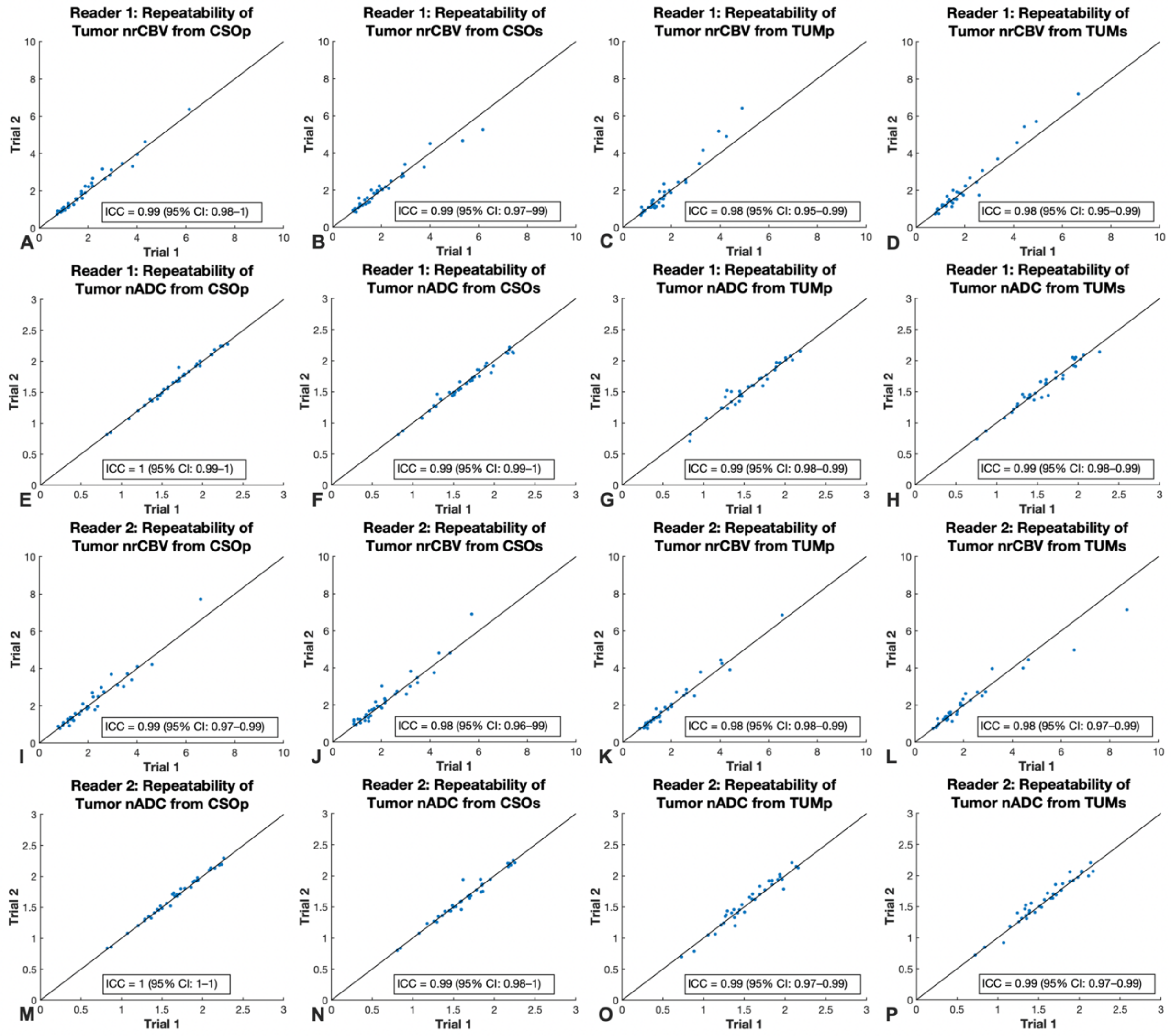

Figure S3. ICC analyses within readers (Trials 1 & 2). Across both trials, nrCBV and nADC from **A/E/I/M**) CSOp, **B/F/J/N**) CSOs, **C/G/K/O**) TUMp, and **D/H/L/P**) TUMs displayed excellent (r>0.9) intra-reader repeatability for both readers. Because nrCBV data were non-normally distributed, all plots of ICC analyses display the non-transformed data points with the appropriate ICC statistical results from the normally distributed data following Box-Cox transformation.

Figure S4.

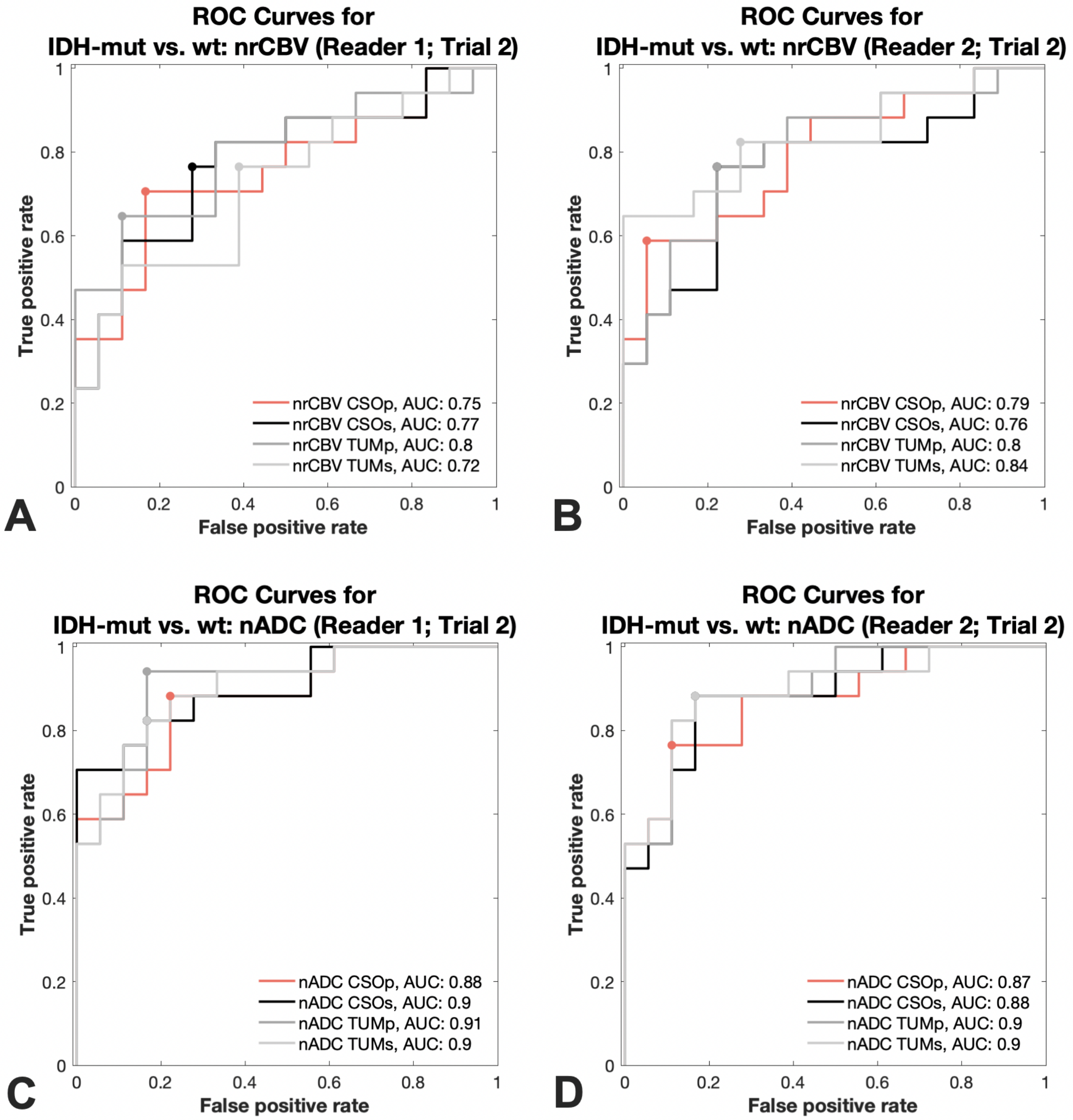

Figure S4. Receiver-operating characteristic (ROC) curves for predicting IDH mutation status (Trial 2). Different normalization methods resulted in similar area under the curve values for IDH-mutation status prediction using **A/B**) nrCBV and **C/D**) nADC in Trial 2, similar to results from Trial 1.

Supplementary Reader Instructions

General:

- Four categories of contralateral NAWM ROI's will be created as part of this study
    1. 3-spheres of 5-mm diameter in <u>contralateral centrum semiovale</u>
        - CSOs.nii.gz
    2. 2-D planar ROI 400-450mm^2 in <u>contralateral centrum semiovale</u>
        - Reader's choice in specific anterior-posterior location
        - CSOp.nii.gz
    3. 3-spheres of 5-mm diameter in <u>slice of the center of tumor</u>
        - TUMs.nii.gz
    4. 2-D planar ROI 400-450mm^2 in <u>slice of the center of tumor</u>
        - Reader's choice in specific anterior-posterior location
        - TUMp.nii.gz
- ITK-SNAP software will be used for ROI segmentations
- All ROI's will avoid cortex, vessels, ventricles, and FLAIR hyperintensity (if the tumor is bilateral)

Requirements:

- ITK-SNAP software
- Timer
- Files for patient's axial anatomical scan, rCBV_reg.nii.gz, and adc_reg.nii.gz

ROI 1: 3-Spheres of 5-mm Diameter in Contralateral Centrum Semiovale

1. Load in 1) anatomical scan, 2) rCBV_reg.nii.gz, and 3) adc_reg.nii.gz
2. Set Segmentation Label to Red Brush (Label 1)
    a. 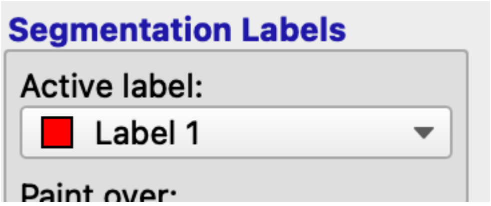

3. In Main Toolbar, select Paintbrush -> Brush Style: Circle -> Brush Size: 5 -> **<u>Brush Options: 3D</u>**
    a. 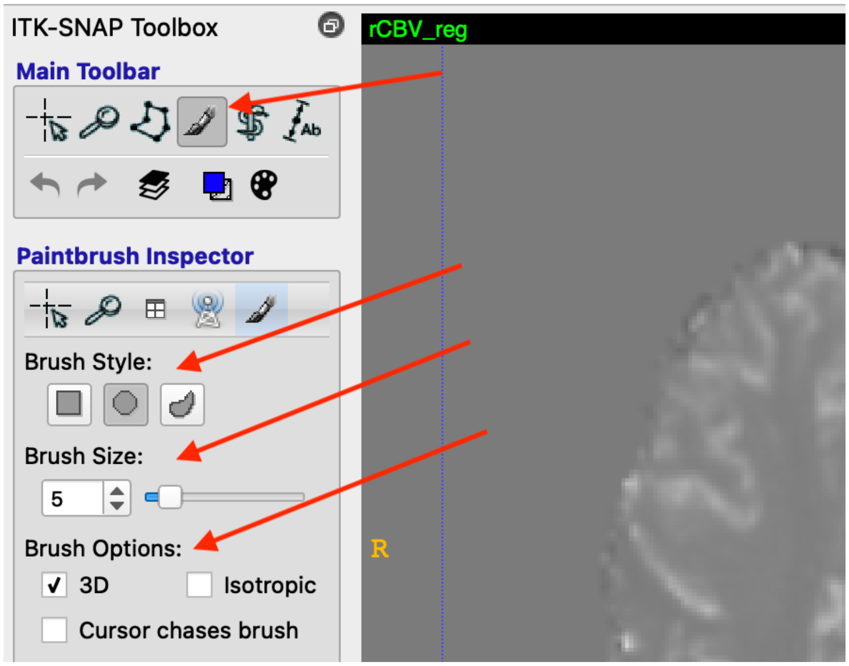

4. Scroll to ~3mm above the superior tip of the lateral ventricles into the centrum semiovale
5. ***Start Timer***
6. Place 3 intra-axial slice spherical ROI's (one-click each) from anterior to posterior: (see images; confirm volume count is 171 mm^3)
    a. Anterior, Middle, Posterior

b. 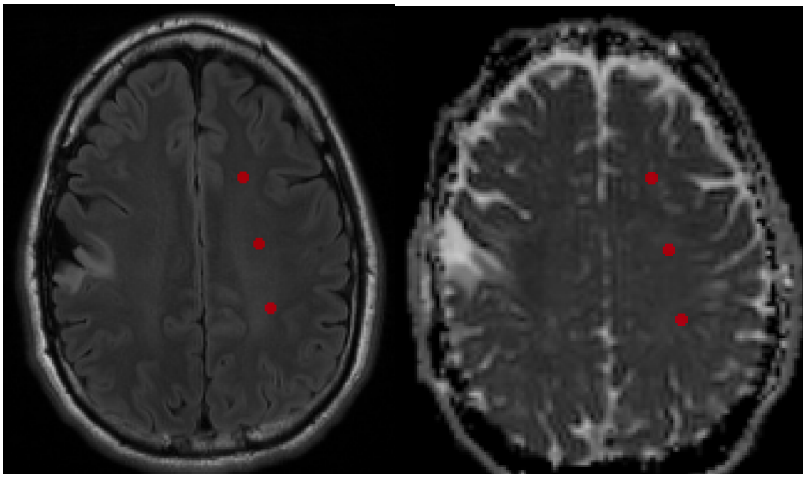

c. 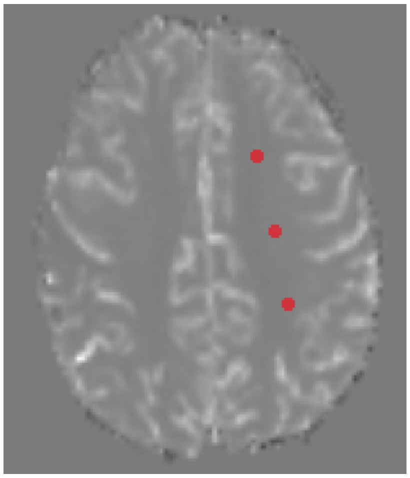

d.

		Label Name	Voxel Count	Volume (mm3)
0	■	Clear Label	12582741	1.258e+07
1	■	Label 1	171	171

7. Save segmentation as **CSOs.nii.gz**
8. ***Stop Timer***

ROI 2: 2-D Planar ROI (Similar to 2cmx2cm) in Contralateral Centrum Semiovale

1. Click Segmentation -> Unload Segmentation to start new segmentation
2. In Main Toolbar, select Paintbrush -> Brush Style: Circle -> Brush Size: 5 -> Brush Options: **De-select 3D**
    a. 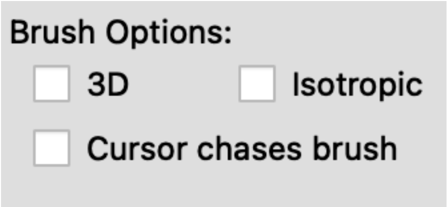

3. Scroll to ~3mm above the superior tip of the lateral ventricles into the centrum semiovale
4. ***Start Timer***
5. Place 2-D freehand ROI of 400-450 mm^2 **(Verify Freehand ROI size using Segmentation -> Volumes and Statistics)** (see images below)
    a. If contiguous ROI cannot be drawn on a single slice, then draw on 2 consecutive slices
    b. 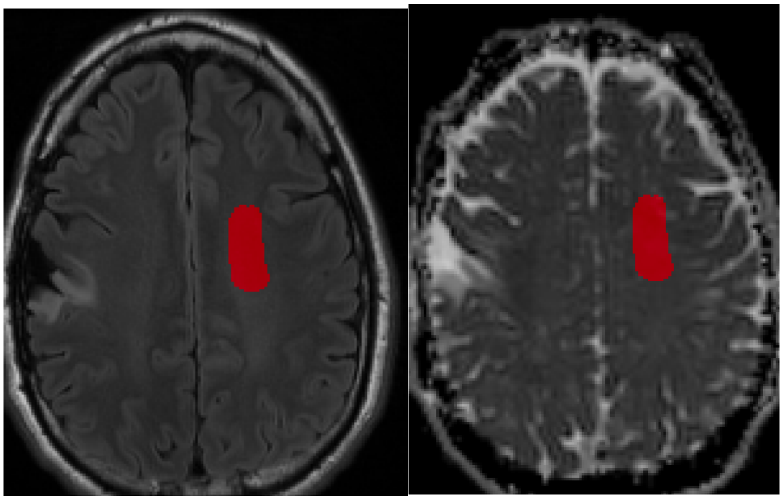

c. 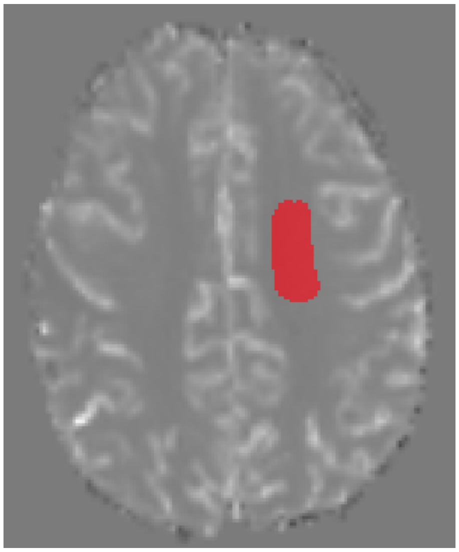

d.

		Label Name	Voxel Count	Volume (mm3)
0	■	Clear Label	12582478	1.258e+07
1	■	Label 1	434	434

6. Save segmentation as **CSOp.nii.gz**
7. ***Stop Timer***

ROI 3: 3-Spheres of 5-mm Diameter in Slice of the Center of Tumor

1. Click Segmentation -> Unload Segmentation to start new segmentation
2. In Main Toolbar, select Paintbrush -> Brush Style: Circle -> Brush Size: 5 -> **Brush Options: 3D**
    a. 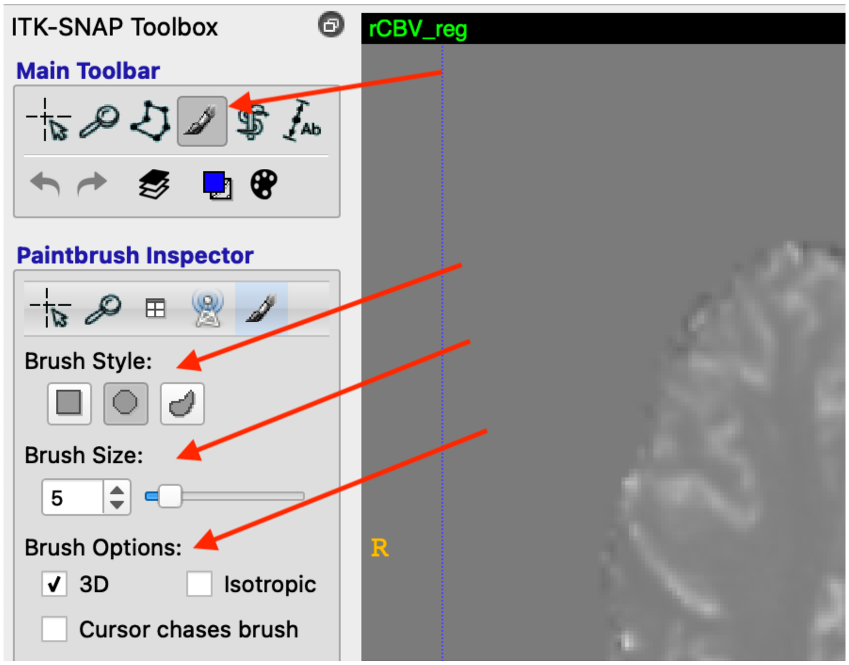

3. Scroll to slice approximately showing the center of tumor
4. ***Start Timer***
5. Place 3 intra-axial slice spherical ROI's (one-click each) from anterior to posterior: (see images; confirm volume count is 171 mm^3)
    a. Anterior, Middle, Posterior

b. 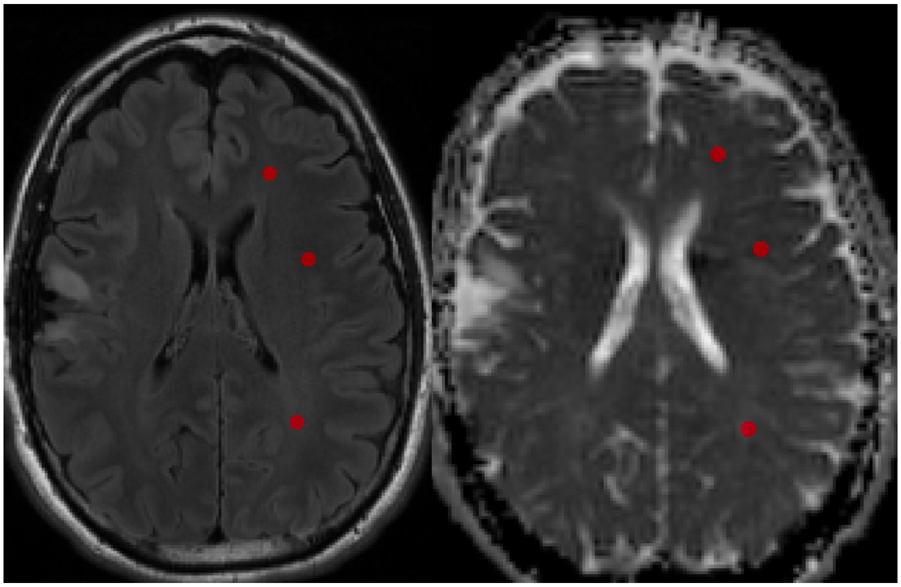

c. 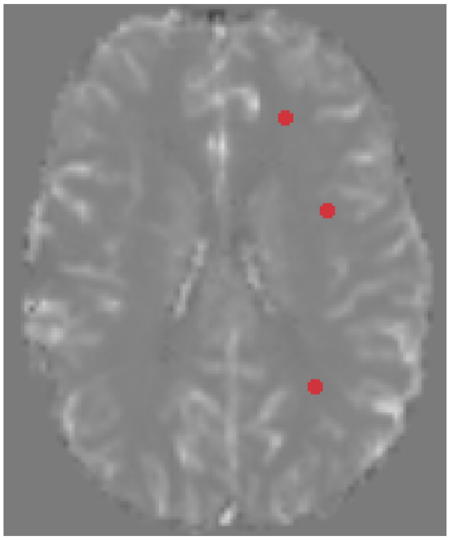

d.

	Label Name	Voxel Count	Volume (mm3)
0	Clear Label	12582741	1.258e+07
1	Label 1	171	171

6. Save segmentation as **TUMs.nii.gz**
7. ***Stop Timer***

ROI 4: 2-D Planar ROI (Similar to 2cmx2cm) in Slice of the Center of Tumor

1. Click Segmentation -> Unload Segmentation to start new segmentation
2. In Main Toolbar, select Paintbrush -> Brush Options: <u>**De-select 3D**</u>
    a. 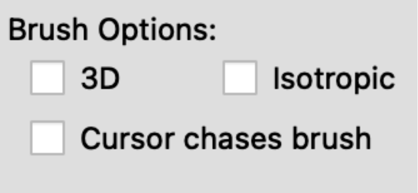

3. Scroll to slice approximately showing the center of tumor
4. ***Start Timer***
5. Place 2-D freehand ROI of 400-450 mm^2 **(Verify Freehand ROI size using Segmentation -> Volumes and Statistics)** (see images below)
    a. If contiguous ROI cannot be drawn on a single slice, then draw on 2 consecutive slices
    b. 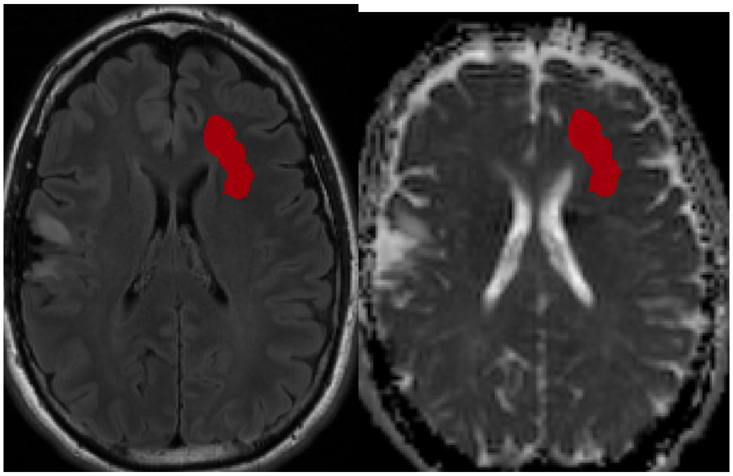
    c. 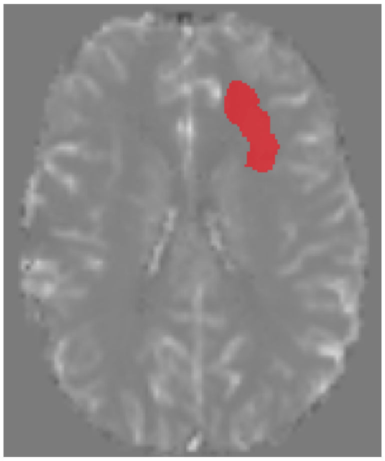

d.

		Label Name	Voxel Count	Volume (mm3)
0	■	Clear Label	12582486	1.258e+07
1	■	Label 1	426	426

6. Save segmentation as **TUMp.nii.gz**
7. ***Stop Timer***
